# Probiotic potential of *Lactiplantibacillus plantarum* LBK from Koumiss in reducing hyperuricemia through gut microbiota modulation

**DOI:** 10.3389/fmicb.2025.1716437

**Published:** 2026-05-20

**Authors:** Panpan Zhao, Yibo Lu, Jianchun Xu, Hui Zhong, Qingqing Yan, Zhifeng Fang, Zhexin Fan, Qin Wang, Yuhui Li, Baokun Li, Xian Wang

**Affiliations:** 1 Key Laboratory of Agricultural Product Processing and Quality Control of Specialty (Co-construction by Ministry and Province), School of Food Science and Technology, Shihezi University, Shihezi, Xinjiang, China; 2 Key Laboratory for Food Nutrition and Safety Control of Xinjiang Production and Construction Corps, School of Food Science and Technology, Shihezi University, Shihezi, Xinjiang, China; 3 Engineering Research Center of Storage and Processing of Xinjiang Characteristic Fruits and Vegetables, Ministry of Education, School of Food Science and Technology, Shihezi University, Shihezi, Xinjiang, China; 4 Innovation Center for Specialty Dairy Products and Processing Technology, XPCC, Shihezi, Xinjiang, Xinjiang, China; 5 Engineering and Technology Research Center of Xinjiang Camel Milk, School of Food Science and Technology, Shihezi University, Shihezi, Xinjiang, China; 6 Karamay Lvcheng Agricultural Development Co., Ltd, Karamay, Xinjiang, China; 7 Institute of Agro-products Processing, Xinjiang Academy of Agricultural and Reclamation Sciences, Shihezi, Xinjiang, China; 8 Institute of Agricultural Quality Standards and Detection Technology, Xinjiang Academy of Agricultural Sciences, Urumqi, Xinjiang, China

**Keywords:** hyperuricemia, Koumiss, *Lactiplantibacillus plantarum*, uric acid transporters, gut microbiota, xanthine oxidase

## Abstract

Hyperuricemia, a metabolic disorder resulting from purine metabolic dysfunction, poses a significant threat to public health. Probiotics, in particular lactic acid bacteria, have the potential to exert therapeutic benefits in the treatment of some metabolic diseases. In this study, 73 lactic acid bacteria were isolated from Koumiss, and their ability to degrade inosine and guanosine, inhibit xanthine oxidase, and other biological properties were assessed through *in vitro* experiments. The finds revealed that *L. plantarum* LBK exhibited a significant inhibition of xanthine oxidase activity compared to other strains. It demonstrated superior adherence to the cell surface and tolerance to acid and bile salt compared to all other strains. Subsequently, the strain with the highest overall performance was selected to investigate its potential to lower uric acid in mice with hyperuricemia. Additionally, compared to the model (MOD) group, uric acid, urea nitrogen, and creatinine levels in serum were significantly decreased in the *L. plantarum* LBK high dose (H-LBK) group. At the same time, *L. plantarum* LBK alleviated the pathological and inflammatory changes of liver and kidney. *L. plantarum* LBK treatment acted on kidney gene expression. It up-regulated the gene expression of ATP binding cassette subfamily G member 2 (ABCG2), and down-regulated the gene expression of both uric acid transporter 1 (URAT1) and glucose transporter 9 (GLUT9). 16S rRNA sequencing analysis revealed the effects of *L. plantarum* LBK treatment on gut microbiota. This treatment decreased the Firmicutes/Bacteroidetes ratio (F/B ratio). It also increased the abundances of *lachnospiraceae_NK4A136_group, Enterobacteriaceae_uncultured, and Parabacteroides.* Besides, non-targeted metabolomic analysis revealed alterations in cysteine and methionine, pyrimidine, vitamin B6, and tryptophan metabolism pathways due to the *L. plantarum* LBK treatment. The treatment also resulted in a reshaping of gut microbial composition and metabolism, ultimately alleviating hyperuricemia in mice. To sum up, *L. plantarum* LBK can improve the health of patients with hyperuricemia by regulating the gut microbiota, providing a relatively safe approach for consumers to alleviate hyperuricemia.

## 1 Introduction

As modern lifestyles and dietary patterns are transformed randomly, the occurrence of hyperuricemia (HUA) has risen rapidly around the world and has become a serious metabolic disease that threatens human health. HUA means the fasting blood uric acid (UA) level exceeding 420 μmol/L within the normal purine diet (Li et al., 2021). High UA content may lead to having gout. Also, HUA is not only the main cause of gout, but relates to common chronic diseases such as hypertension, chronic kidney disease, diabetes, and cardiovascular disease (Liu et al., 2018).

Uric acid is the final product of human purine catabolism (James et al., 2021). The final three-step degradation is the key part, in which purine nucleoside is dephosphorylated to form a purine base, which is then converted into xanthine under the action of xanthine oxidase (XOD), and then oxidized into UA by XOD (Chen et al., 2016). Approximately 70% of UA is excreted in urine, and 30% is eliminated through the gastrointestinal tract (Lipkowitz, 2012). Excessive production and insufficient excretion of UA are the main reasons for increased blood uric acid. In most mammals, uric acid is converted into soluble allantoin by uric acid oxidase and excreted through the kidney. However, in the early stage of ancient human evolution, the uricase gene gradually mutated, resulting in the complete loss of uricase function (Kratzer et al., 2014). The accumulation of UA leads to the formation of urate, which may cause great pain and inflammatory reaction, leading to liver and kidney damage, and cannot be relieved spontaneously with time (Aroor et al., 2017). At present, diet management and taking medicine are the main methods to alleviate hyperuricemia. However, it is difficult to balance nutrient intake by controlling purine intake in diet (James et al., 2021) and there are obvious side effects when taking medicine (van der Klauw et al., 1994). Given the limitations of current treatment approaches, there is a significant practical need to identify and establish an innovative, effective method for treating HUA.

The gut microbiota plays a crucial role in disease treatment, and the exploration of probiotics for the management of HUA is garnering increasing attention (Wang et al., 2022). Probiotics are an integral component of the native human gut microbiota (Ma et al., 2024). Lactic acid bacteria (LAB) are common probiotics in the intestinal tract of the host. In addition to promoting intestinal health, LAB have immunomodulatory, anti-allergic, and blood pressure, lipid, and glucose regulatory functions (Martínez-Cañavate et al., 2009; Wang et al., 2021; Wei B. et al., 2022; Wei J. et al., 2022). In recent years, various studies have demonstrated that probiotics, especially LAB, may affect UA levels, inflammatory mediators, gut microbiota, and purine metabolism in the host *in vivo*, which may directly or indirectly result in HUA (Cao et al., 2022a; Cao et al., 2022b). Some reports were published on the absorption of purine substances *in vitro*, thereby reducing the intestinal absorption of purine substances and lowering UA in rats (Yamada et al., 2017; Yamanaka et al., 2018). Similarly, some LAB strains showed their unique enzymatic activity of hydrolyzing purine (Wang et al., 2019; Kuo et al., 2021).

However, studies on the interventional effects of uric acid lowering lactic acid bacteria on fecal metabolites in hyperuricemia mice are relatively scarce. The novelty of our study lies in the addition of analyses on HUA mice fecal metabolites and short-chain fatty acid (SCFA) profiles, as well as the correlation study between the gut microbiota and the fecal metabolome, aiming to reveal the potential mechanism for alleviating hyperuricemia.

Koumiss, a traditional fermented dairy product, is known to produce a substantial amount of LAB. In regions where Koumiss is commonly consumed, it is regarded as a health-enhancing beverage that enhances metabolism and maintains the health of the neurological system and abdominal glands (Afzaal et al., 2021). Initially, we investigated the degradation capabilities of inosine and guanosine in potential LAB candidates and conducted a systematic evaluation of their biological properties *in vitro*. Subsequently, to investigate the effects of the candidate LAB *in vivo*, we established a HUA mouse model (induced by potassium oxonate and a high-purine diet). Finally, the impact of *L. plantarum* LBK on the physiological indicators, gut microbiota, and fecal metabolite were thoroughly analyzed. This study was aimed at screening LAB from Koumiss with anti-hyperuricemia ability *in vitro* and *in vivo* and explore the underlying mechanisms involved. These findings provided heoretical references and valuable approaches to mitigating hyperuricemia.

## 2 Materials and methods

### 2.1 Materials and reagents

The Koumiss samples were collected from the Yili region of Xinjiang, China. MRS broth medium and MRS solid medium were purchased from Qingdao Haibo Biotechnology Co., Ltd. (Qingdao, China). PBS buffer solution and XOD was purchased from Solarbio Science and Technology Co., Ltd. (Beijing, China). Inosine, guanosine, and CMC-Na were supplied by Sigma Aldrich Co. Ltd. (St. Louis, MO, USA). Allopurinol fructose and RNA (from yeast) have been purchased from Shanghai Meikelin Biochemical Technology Co., Ltd. (Shanghai, China). Yeast extract was purchased from Angel Yeast Co., Ltd. (Yichang, China). Commercial kits such as UA, creatinine (CRE), urea nitrogen (BUN), and XOD were obtained from Nanjing Jincheng Bioengineering Institute (Nanjing, China). Quantitative real-time PCR kits were sourced from Beijing Cowin Biotech Co., Ltd. (Beijing, China). Tumor necrosis factor-*α* (TNF-α) and interleukin-6 (IL-6) commercial ELISA assay kits were purchased from Hangzhou Lianke Biotechnology Co., Ltd. (Hangzhou, China).

### 2.2 Isolation and cultivation of LAB

Koumiss samples were immediately stored at −80 °C until analysis. The LAB strains isolated by means of the pour plate method. A mixture of 1 mL Koumiss and 9 mL sterile saline was prepared. Serial dilutions were then created, which were subsequently plated on MRS agar and incubated at 37 °C for a period of 48 h. Following this, isolates that were Gram-positive, rod-shaped, and catalase-negative were selected and purified through multiple successive transfers. The purified LAB strains were then inoculated in 60% glycerol and stored at −80 °C within a refrigerator.

### 2.3 Screening of inosine and guanosine degrading LAB strains

The *in vitro* degradation capabilities of the strains for inosine and guanosine were assessed using a method (Li, 2014) previously reported with slight modification. A total of 73 strains of LAB were isolated from Koumiss for testing. The strains were then washed three times with PBS (0.1 mol/L, pH 7.0), resuspended with 750 μL of 1.26 mM inosine-guanosine solution, and incubated for 120 min at 37 °C and 120 r/min. Later, the solution was centrifuged at 6000 rpm for 10 min to obtain the supernatant, and the HClO_4_ solution (0.1 mol/L) was added to terminate the reaction. Subsequently, the HClO4 solution (0.1 mol/L) was added to stop this reaction. Residual inosine and guanosine were determined in solution using a high-performance liquid chromatography (HPLC) system as detailed below.

A 20 μL aliquot of the filtered solution was taken for HPLC analysis (LC-20A, Shinadzu Corporation, China; chromatographic column, Spursil C18, 4.6 × 250 mm, 5 mm; column temperature, 30 °C; UV detection wavelength, 254 nm; mobile phase, 20 mmol/L KH_2_PO_4_ (pH 3.0): Methanol = 99:1; elution time, 45 min; flow rate, 1 mL/min). Using the following formula to calculate the inosine and guanosine degradation capacity of the isolate (Equation 1).

D(100%)=[(C−X)/C]×100 (1)

where D represented the degradation rate of inosine or guanosine, and C and X represented the levels of inosine and guanosine in the supernatant after reaction of the bacteria-free and bacteria-containing strains, respectively.

### 2.4 XOD inhibitory activity (XOI) assay

The XOI of samples was determined following the method by (Chen et al., 2017). The collected isolated cells were resuspended in PBS (0.1 mol/L, pH = 7.5). The bacterial suspension was cultured at 37 °C for 16 h, then centrifuged at 6000 r/min for 10 min, and the supernatant was obtained (cell metabolites). The bacterial suspension was undergone ultrasonic treatment, followed by centrifugation at 6000 r/min for a duration of 10 min and taking supernatant to obtain cell contents. The enzymatic reaction system was prepared in accordance with Table 1. The inhibition of XOD by the strain was calculated with reference to the following method (Equation 2).

XOI(%)=[1−(ODa−ODb)/(ODc−ODd)]×100 (2)

**Table 1:** The reaction system of xanthine oxidase inhibitory ability.

Groups	PBS buffer (μL)	XOD (μL)	Sample (μL)	Xanthine (μL)
a	0	50	50	100
b	50	0	50	100
c	50	50	0	100
d	100	0	0	100

Where a is the positive experimental group with the addition of samples and enzymes; b is a positive control group with no enzyme added to the sample; c is the negative experimental group with no sample added with enzyme; d is the negative control group without the addition of enzymes and samples.

### 2.5 Biological properties of LAB

The tolerance of LAB to low pH conditions and high concentrations of bile salts was investigated as stated by Mulaw et al. (2019) with modifications. The low pH resistance of was evaluated by inoculating the bacterium into phosphate buffer solution (PBS, pH 2.0 and 3.0) at 37 °C for 3 h; the tolerance to bile salts was assessed by inoculating the bacterium into PBS containing 0.1 and 0.3% bile salts for 4 h.

The self-aggregation ability and hydrophobicity of the strains were determined according to a previously published method (Mulaw et al., 2019) with slight modification. To examine the ability of self-aggregation, we adjusted the absorbance value of the bacterial suspension to about 0.8 (H_0_) at 600 nm and cultured at 37 °C. After 3, 6, and 24 h, the absorbance was measured (H_t_). Bacterial cells were meticulously washed using PBS buffer and subsequently resuspended in an identical buffer. The absorbance was then adjusted to a value of 1.0 (A_0_) at a wavelength of 600 nm. Subsequently, we combined 1 mL of xylene and 3 mL of suspension by vortexing for 1 min. Following a 15-min incubation period at room temperature, the aqueous phase was removed, and its absorbance was measured (At). The results were presented as percentages in accordance with the following formula (Equations 3−4):

$$\text{Self}-\text{aggregation ability}\;(\%)=[1-(H_{t}/H_{0})]\times 100$$ (3)

$$\text{Hydrophobicity}\;(\%)=[1-(A_{t}/A_{0})]\times 100$$ (4)

### 2.6 Identification of candidate LAB

The DNA of candidate LAB was extracted, and PCR amplification was performed with universal primers 27F (AGAGTTTGATCCTGGCTCAG) and 1492R (GGTTACCTTGTTACGACTT). The PCR products of selected strains were sequenced by Sangon Biotech Company, Shanghai, China. The obtained sequences were matched on NCBI, and a phylogenetic tree was constructed by MEGA 11.0.

### 2.7 Animal treatment

Forty Balb/c male mice (18 ± 2 g) were obtained from Henan Sike Beisi Biotechnology Co., Ltd. with the license number SCXK (Yu) 2020–0005. The experimental protocol received approval from the Animal Care Committee of the Shihezi University Biology Ethics Committee, China (A2023-090).

The mice were randomly assigned to 5 distinct groups: the control (CON) group, the model (MOD) group, *L. plantarum* LBK high dose (H-LBK) group, *L. plantarum* LBK low dose (L-LBK) group, and allopurinol (ALLO) group. According to the reported method (Cao et al., 2022a; Cao et al., 2022b) with some modifications, the CON group was provided with a standard diet and water, while the other groups were subjected to a high-purine diet (comprising 400 g/kg yeast extract, 20 g/kg RNA, and 5% fructose water). To effectively establish a hyperuricemia model, the mice were given 350 mg/kg of potassium oxonate in 0.5% CMC-Na solution during 28 days. From the third week, the MOD and CON groups were given sterile saline, while the H-LBK group was given 10^9^ CFU/mL of *L. plantarum* LBK, the L-LBK group was gavaged 10^7^ CFU/mL of *L. plantarum* LBK, furthermore, the ALLO group was administered 5 mg/kg of allopurinol. After 4 weeks, mice blood, kidney, liver, and intestinal contents were collected and stored at −80 °C for further analysis.

### 2.8 Body weight and organ coefficient measurement

The body weight of the mice was monitored and recorded three times. Additionally, the weight of the entire kidney and liver was recorded, and subsequently, the organ index was determined utilizing the provided formula (Equation 5).

$$\text{Origan index}(\%)=(M_{\text{Organ}}/M_{\text{Body}})\times 100$$ (5)

### 2.9 Serum biochemical analysis

The levels of UA, CRE, and BUN in the mice’s serum were assayed in accordance with the instructions provided in the kit.

### 2.10 Histopathological assessment

After dissection, the liver and kidney samples were excised from the abdominal cavity of the mice, the liver and kidney weights were recorded. Subsequently, the liver and kidney samples were fixed in a 10% formaldehyde phosphate buffer solution for a duration of 24 h. Following fixation, the tissues were embedded in paraffin, and 5 μm serial sections were prepared for hematoxylin and eosin (H&E) staining (Wuhan Service Biotechnology Co., Ltd., China) to evaluate histopathological alterations.

### 2.11 Assessment of inflammatory

The liver and kidney tissues of the mice were homogenized while kept on ice, and then centrifuged at 3500 rpm for 10 min. The resulting supernatant was subsequently collected. The concentration of IL-6 and TNF-*α* were determined using commercial ELISA assay kits.

### 2.12 Relative expression of UA transporter in kidney

Trizol reagent was employed to extract RNA. Total RNA was reverse transcribed into cDNA utilizing the Prime Script™ RT master mix kit. Subsequently, the expressions of the mRNA encoding uric acid transporter 1 (URAT1), glucose transporter 9 (GLUT9), and ATP-binding cassette subfamily G member 2 (ABCG2) were quantified using a Step One Plus real-time PCR system (Applied Biosystems, USA) following the instructions provided in the ChamQ SYBR qPCR master mix kit. GAPDH gene served as an endogenous control for the assay. The specific forward and reverse primers employed in this process are detailed in Supplementary Table S1.

### 2.13 16S rRNA gene sequencing of gut microbiota

Fecal samples were collected and immediately frozen at −80 °C on the last day of gavage administration. Illumina MiSeq sequencing for fecal samples was carried out according to previously reported procedures (Chen S. et al., 2018; Magoč and Salzberg, 2011; Stackebrandt et al., 1994). QIAamp DNA Stool Mini Kit was used to extract the total DNA of contents, and for the amplification of the microbial V3-V4 region via PCR, the primers 338F (5′-ACTCCTACGGGAGGCAGCAG-3′) and 806R (5′-GGACTACHVGGGTWTCTAAT-3′) were utilized. Sequencing was carried out utilizing the Illumina Miseq PE300/NovaSeq PE250 platform. (Wuhan Biowefind Co., Ltd., Wuhan, China).

### 2.14 Fecal untargeted metabolomics analysis

A 400 μL extraction solution, composed of methanol and water in a ratio of 4:1 (v/v) and containing 0.02 mg/mL of the internal standard (L-2-chlorophenylalanine), was employed for metabolite extraction. The sample solution was ground in a cryogenic tissue grinder for 6 min at −10 °C and 50 Hz, subsequently followed by a low-temperature ultrasonic extraction step for 30 min, maintained at 5 °C and 40 kHz.

The samples were centrifuged at 13000 × g for 15 min at 4 °C, and the supernatant was transferred to an injection vial equipped with an in-line tube for direct analysis on the machine. The LC–MS analysis was performed using an Ultra-high Performance Liquid Chromatography tandem Fourier Transform Mass Spectrometry UHPLC – Q Exactive HF-X system (Thermo Fisher Scientific, USA). The raw LC–MS data were processed using the metabonomic software Progenesis QI (Waters Corporation, Milford, USA). Concurrently, the MS and MS/MS spectrometry information was cross-referenced with metabolic public databases, including HMDB^1^ and METLIN^2^. The processed matrix data were uploaded to the MajorBio-Cloud Platform^3^ for further analysis. The differences between the preprocessed matrix files were examined. The differential metabolites were annotated using the KEGG database^4^ to identify the pathways in which they participated.

### 2.15 Statistical analysis

The data were represented as the mean with standard deviation (±SD). For comparisons between two groups, unpaired two-sided Student’s *T*- tests were employed. For datasets with more than two groups, one-way ANOVA and two-way ANOVA were utilized for statistical analysis. All statistical assessments were conducted using GraphPad Prism version 9.0, with statistical significance set at *p* < 0.05. Each experimental result was replicated at least three times to ensure reproducibility and reliability.

## 3 Results

### 3.1 Screening of LAB strains degrading inosine and guanosine

To identify potential probiotics for screening purposes, we assessed the degradation capacity of LAB strains isolated from Koumiss for inosine and guanosine via HPLC. The results showed that most of the LAB strains had purine nucleoside degradation ability and the degradation rates of these strains ranged from 0.17 to 100% (Supplementary Table S2). Among the studied samples, the degradation rates of 1–3, 1–2, 2–1, 25–14, 11–3, 29B, 1 M23, and 9A were notably higher than those observed in other LAB within a 2 h period. Therefore, we selected these eight LAB strains for further study.

### 3.2 The XOD inhibition ability of LAB

**Figure 1:**
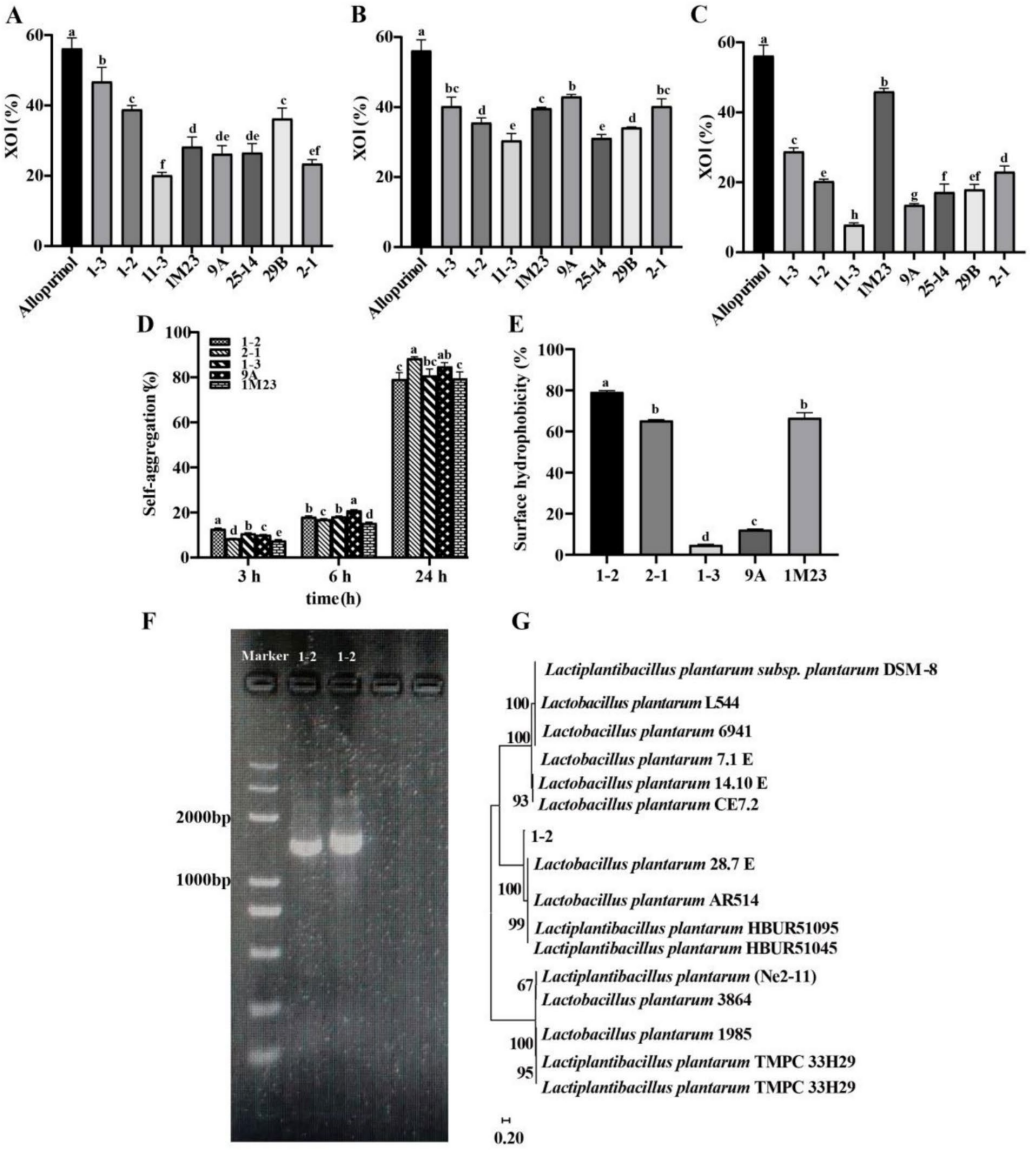
Biological properties of isolated strains. **(A)** XOI of cell suspension, **(B)** XOI of cell-free content, **(C)** XOI of cell metabolites, **(D)** Self-aggregation, **(E)** Surface hydrophobicity, **(F,G)** Identification of the strain 1–2. Different lowercase letters denote significant differences (*p* < 0.05).

XOD is a key enzyme in uric acid synthesis and can reduce UA levels by inhibiting xanthine oxidase activity. Consequently, the capacity of the eight isolates to inhibit the activity of XOD was assessed and the result was depicted in Figures 1A–C. Allopurinol demonstrated a superior capacity to inhibit XOD, exhibiting a significantly higher inhibition rate of 56.17% compared to LAB. The inhibition of XOD by various LAB strains demonstrated significant variation. Notably, the cell suspension and cell-free content of 1–3 exhibited the highest inhibition rates. In contrast, the inhibition rates of XOD by cell-free content of 11–3 and 25–14 were significantly lower than those of the other six strains (*p* < 0.05). In terms of the XOD inhibition ability of strain content, 9A had the strongest inhibition ability. Compared with other groups, the strain metabolites of 11–3 had the weakest XOD inhibition ability (*p* < 0.05). Therefore, the six strains (1–3, 1–2, 1 M23, 9A, 29B, and 2–1) will be chosen for further investigation in subsequent experiments.

### 3.3 Biological properties of LAB

Tolerance to low pH conditions and bile salts for a certain period is one of the important criteria for selecting probiotics, which provides a guarantee for the survival of the strain in the intestine and plays an active role. We tested the low pH resistance (pH = 3) and bile salt resistance (0.1, 0.3%) of the strains. The results are shown in Table 2. After being treated at low pH for 3 h, all six strains could survive, and the survival rates were over 50%, among which the survival rate of 2–1 was as high as 92.00%. The viability of these six strains varied to bile salt, with the highest survival rate observed at 2–1 in both 0.15 and 0.3% bile salt, achieving 81.00 and 61.65%, respectively.

**Table 2:** The survival rate of strains under low pH and bile salts.

Strains	Survival rate (%)		
	pH = 3	0.15% bile salts	0.3% bile salts
1–2	87.75 ± 0.15^b^	72.95 ± 4.75^a^	43.89 ± 1.03^c^
29B	85.17 ± 0.15^c^	–	–
1–3	82.17 ± 0.03^d^	71.17 ± 10.11^ab^	37.81 ± 2.04^c^
9A	51.03 ± 0.15^g^	61.14 ± 4.68^c^	43.35 ± 2.27^c^
1 M23	67.17 ± 0.03^f^	76.52 ± 4.99^a^	54.38 ± 5.6^b^
2–1	92.00 ± 0.03^a^	81.00 ± 8.45^a^	61.65 ± 6.08^bc^

Different lowercase letters denote significant differences (*p* < 0.05).

The self-aggregation ability and hydrophobicity of the bacterial surface, the characteristics of LAB, can influence its ability to absorb other bacteria and intestinal epidermal cells. As illustrated in Figure 1D, with the increase of culture time, there was a notable enhancement in the self-aggregation ability of the strains. The self-aggregation rate consistently exceeded 75% after 24 h. Remarkably, 2–1 exhibited a significantly higher self-aggregation rate than its counterparts, registering an impressive 88.45%. As shown in Figure 1E, the adsorption capacity of different LAB strains for xylene varied, and there was a great variability between the hydrophobicity capacity of these LAB strains. The hydrophobicity of 1–2 for xylene was 79.17%, the hydrophobicity of 1 M23 was 64.97%, and the hydrophobicity of the other two strains of LAB was below 40%.

Based on these results, the strain of 1–2, characterized by better biological properties and higher efficiency in inosine and guanosine degradation, was selected for further study of 16S rDNA gene analysis. As seen in Figures 1F,G, the strain was identified as *Lactiplantibacillus plantarum* and was named *Lactiplantibacillus plantarum* LBK.

### 3.4 *Lactiplantibacillus plantarum* LBK exerted protective effects in hepatorenal injury

**Figure 2:**
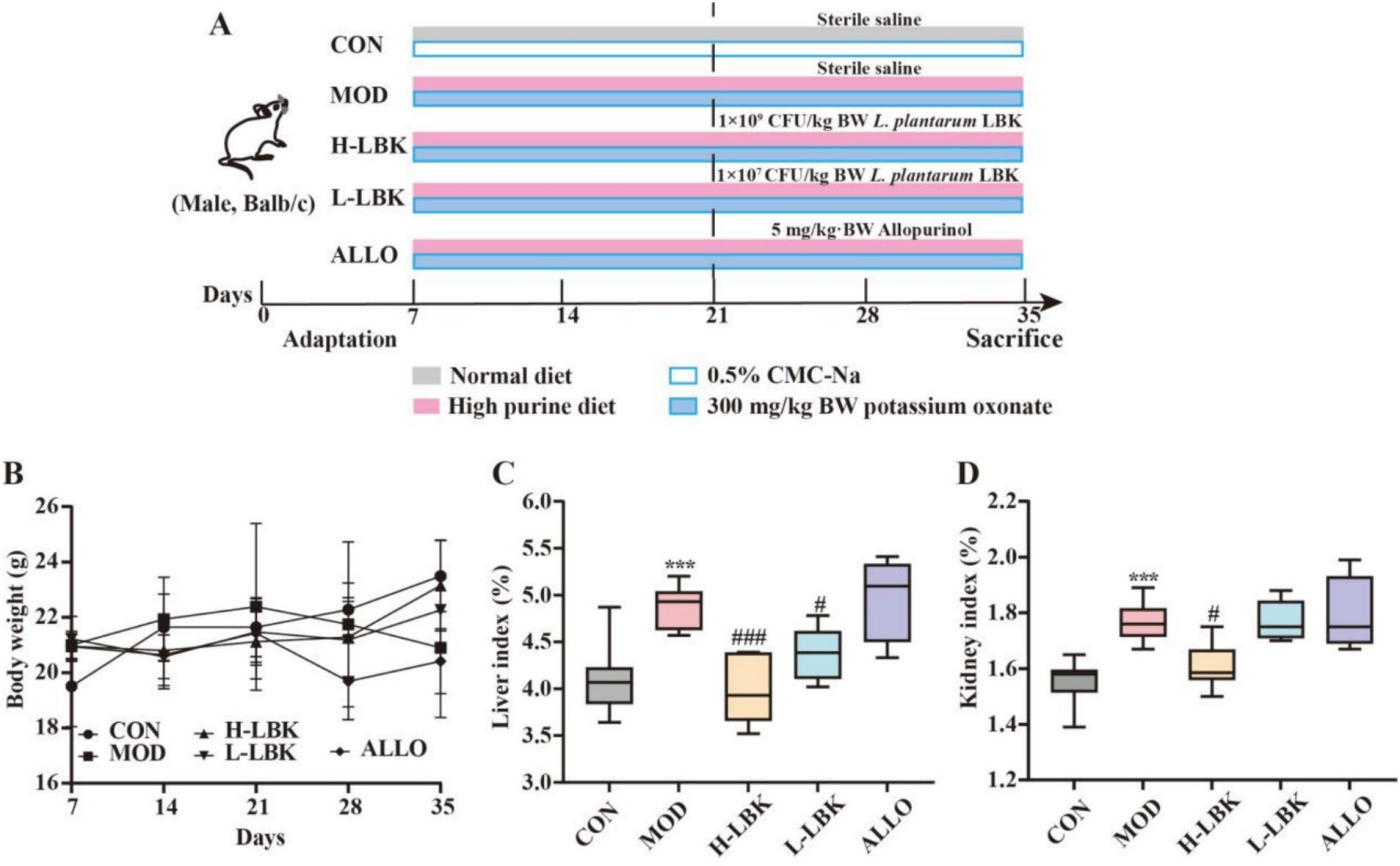
Effect of *L. plantarum* LBK on body weight and visceral coefficient in mice. **(A)** Establishment and treatment stage of HUA mice, **(B)** Body weight, **(C)** Liver index, **(D)** Kidney index. *p < 0.05, **p < 0.01, ***p < 0.001, ****p < 0.0001 vs. CON group. #*p* < 0.05, ##*p* < 0.01, ###*p* < 0.001, ####*p* < 0.0001 vs. MOD group.

The organ index can reflect the overall health status of mice to a certain extent. Atrophy and degeneration of organs will lead to a decrease in organ index, and edema or proliferation of organs will lead to an increase in organ index. Figure 2B showed the body weight of the mice. The weight of mice in the CON group exhibited a gradual increase, while those in the MOD group demonstrated a decrease within 2 weeks post-experiment. The intervention involving *L. plantarum* LBK mitigated the weight loss induced by modeling. As presented in Figures 2C,D, compared to the CON group, the MOD group exhibited significantly elevated liver and kidney indices (*p* < 0.05). This showed that HUA damaged the liver and kidney of mice, and *L. plantarum* LBK can repair this damage.

### 3.5 *Lactiplantibacillus plantarum* LBK reduced the levels of serum UA, CRE, and BUN in hyperuricemia mice

**Figure 3:**
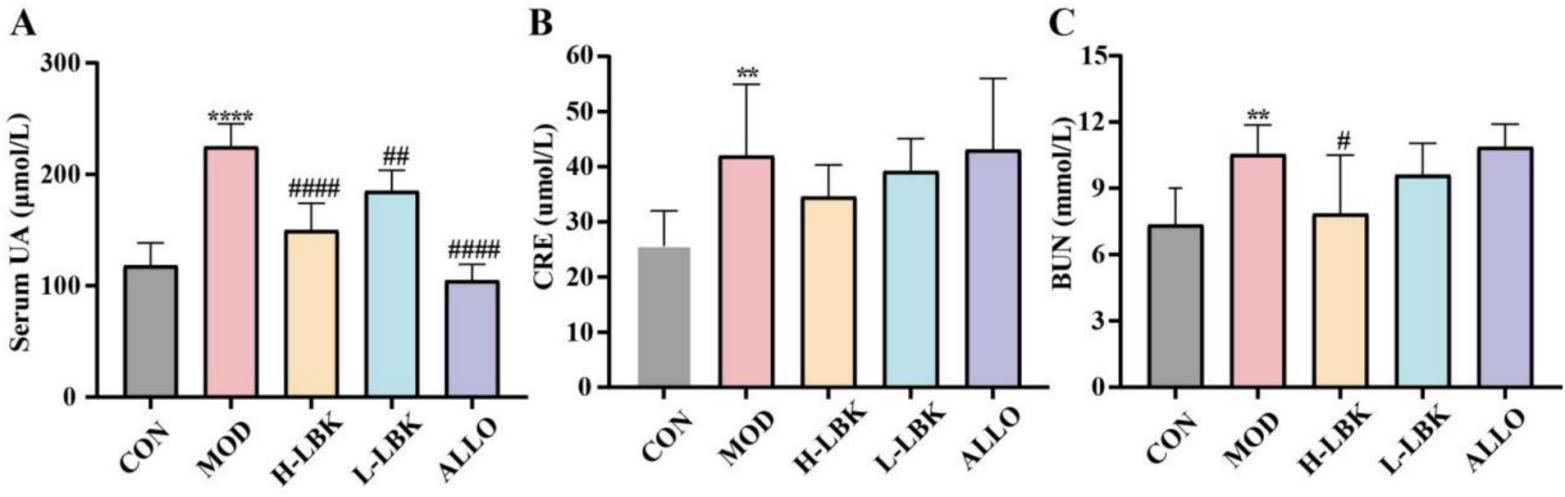
*Lactiplantibacillus plantarum* LBK reduced the levels of serum UA, CRE, and BUN in hyperuricemia mice. **(A)** Serum UA, **(B)** CRE, **(C)** BUN. *p < 0.05, **p < 0.01, ***p < 0.001, ****p < 0.0001 vs. CON group. #*p* < 0.05, ##*p* < 0.01, ###*p* < 0.001, ####*p* < 0.0001 vs. MOD group.

To investigate the effect of *L. plantarum* LBK on serum index levels in hyperuricemia mice, we induced hyperuricemia by gavage of potassium oxonate and the addition of a high-purine diet (Figure 2A). The changes in serum UA, CRE, and BUN of mice across various experimental groups were depicted in Figures 3A–C. As expected, mice in the MOD group exhibited high levels of serum uric acid, and the serum uric acid levels in mice in the MOD group were found to be significantly higher than those in the CON group, which received a normal diet (*p* < 0.01), suggesting that the hyperuricemia mouse model was successfully established. Allopurinol significantly reduced serum UA to healthy levels compared to the CON group, and the analysis of serum samples from the H-LBK group demonstrated no discernible difference in UA levels. It indicated that the intake of 14 days of high-dose *L. plantarum* LBK restored the homeostasis of serum uric acid. Furthermore, treatment with a high dose of *L. plantarum* LBK resulted in a 17.7% reduction in serum creatinine and a 25.62% reduction in BUN. However, the administration of allopurinol did not ameliorate the augmentation of creatinine and urea nitrogen levels induced by hyperuricemia. Notably, the results demonstrated that the high-dose treatment was more effective than the low-dose treatment.

### 3.6 *Lactiplantibacillus plantarum* LBK modulated hepatorenal injury and abolished the increase of proinflammatory cytokines

**Figure 4:**
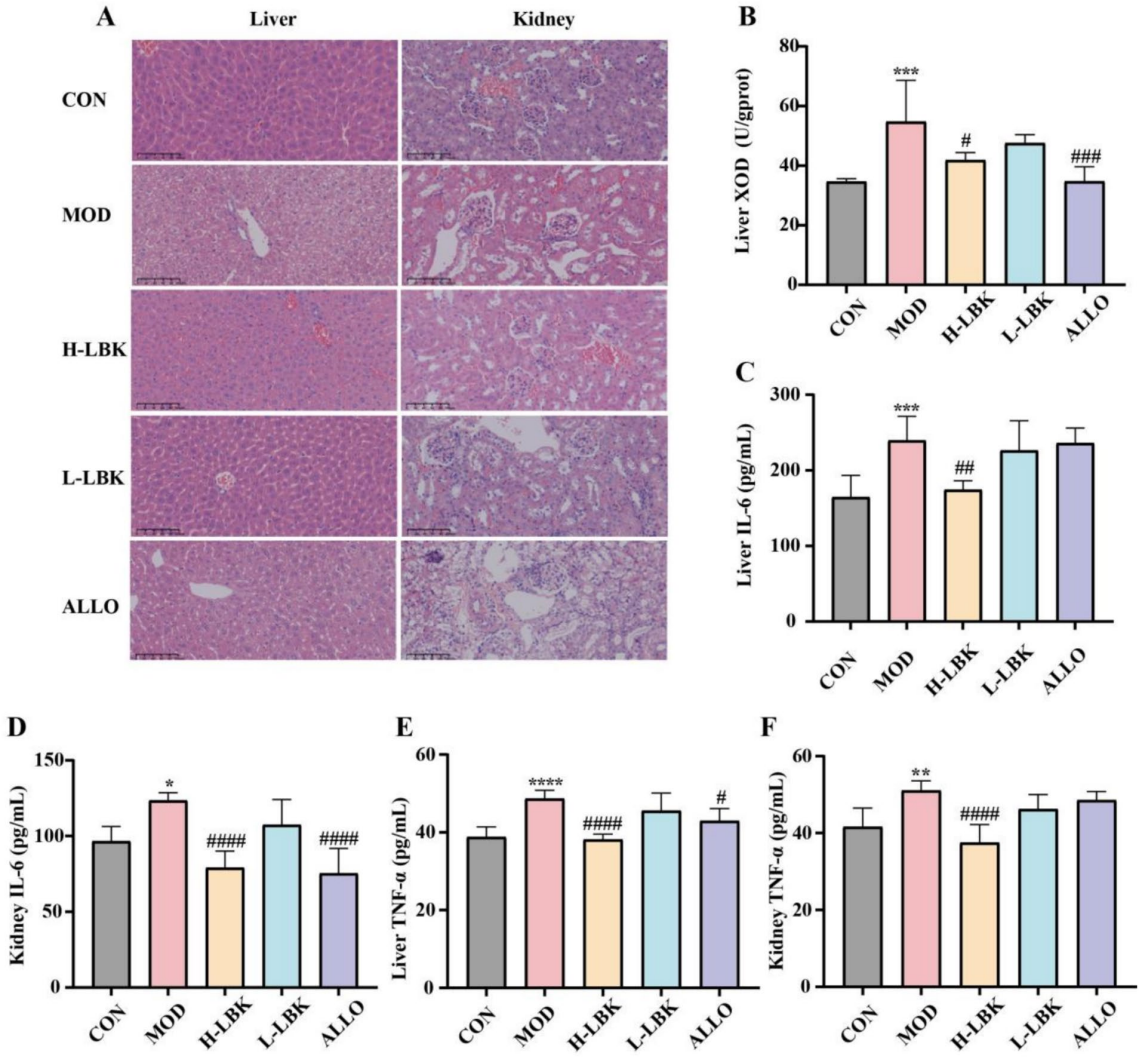
Effect of *L. plantarum* LBK on hepatorenal injury and multiple inflammation cytokines. **(A)** Representative photomicrographs of H&E staining of mice hepatic and renal tissue (H&E 20 ×), **(B)** Liver xanthine oxidase levels, **(C)** Liver interleukin (IL-6) levels, **(D)** Renal interleukin (IL-6) levels, **(E)** Liver tumor cell necrosis factor-*α* (TNF-α) levels, **(F)** Renal tumor cell necrosis factor-α (TNF-α) levels. *p < 0.05, **p < 0.01, ***p < 0.001, ****p < 0.0001 vs. CON group. #*p* < 0.05, ##*p* < 0.01, ###*p* < 0.001, ####*p* < 0.0001 vs. MOD group.

Histopathological examination can visualize the severity of hyperuricemia. The results of H&E staining are presented in Figure 4A. Hepatocytes in the MOD group were found to have more severe hepatic edema along with inflammatory cell infiltration, whereas hepatocytes in the H-LBK group were resembled those of the CON group, with the structure of hepatocyte cords intact. The histological examination of renal tissue revealed a clear deterioration of the glomeruli and tubules in both the MOD and ALLO groups. However, the adverse effects were significantly attenuated by the administration of high-dose *L. plantarum* LBK. The administration of allopurinol did not ameliorate the renal injury observed in HUA mice; instead, it escalated the pressure on renal excretion and exacerbated the severity of the injury. These observations suggested that *L. plantarum* LBK attenuated UA-induced liver and kidney injury.

The impact of *L. plantarum* LBK on anabolic enzyme activities of UA in HUA mice was investigated in conjunction with the activity of liver XOD. As shown in Figure 4B, the hepatic XOD activities exhibited a decrease in mice subjected to *L. plantarum* LBK treatment compared to the MOD group. The activity of XOD in hypouricemic mice was significantly inhibited upon the intragastric administration of a high dose of *L. plantarum* LBK (*p* < 0.05). The activity of XOD in the ALLO group was found to be 36.60% lower than that of the MOD group (*p* < 0.05).

To evaluate the level of inflammatory cytokines, the levels of IL-6 and TNF-*α* in the liver and kidney were detected by ELISA. As illustrated in Figures 4C–F, the concentrations of various proinflammatory factors in the MOD group were demonstrably higher than those of the CON group. Fortunately, *L. plantarum* LBK relieved the situation by lessening TNF-α, and IL-6 compared with that of the MOD group. It is noteworthy that the H-LBK group effectively modulated the levels of inflammatory cytokines in both the liver and kidney, demonstrating a superior anti-inflammatory effect compared to the L-LBK group.

### 3.7 *Lactiplantibacillus plantarum* LBK exerted a positive effect on the expression of renal UA transporters

**Figure 5:**
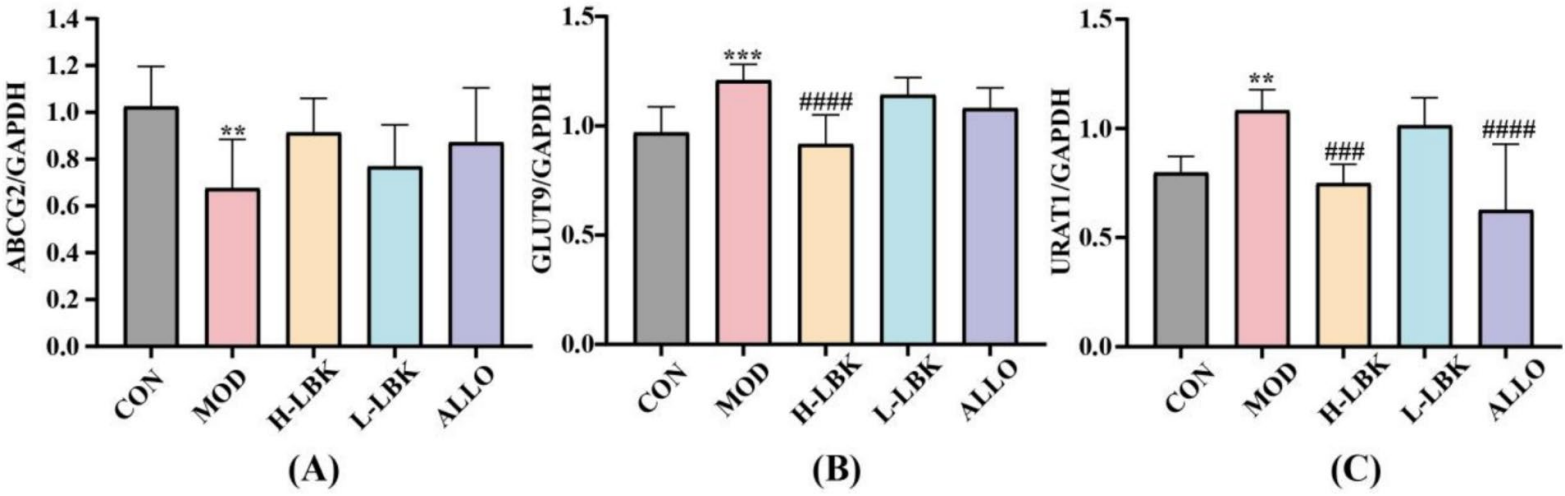
Renal UA transporters expression. **(A)** ATP Binding Cassette Subfamily G Member 2 (ABCG2) **(B)** Glucose Transporter 9 (GLUT9), **(C)** Uric Acid Transporter 1 (URAT1). *p < 0.05, **p < 0.01, ***p < 0.001, ****p < 0.0001 vs. CON group. #*p* < 0.05, ##*p* < 0.01, ###*p* < 0.001, ####*p* < 0.0001 vs. MOD group.

As shown in Figures 5A–C, in comparison to the CON group, the mRNA expression levels of ABCG2 were notably downregulated in the MOD group (*p* < 0.05), while the URAT1 level was significantly increased. *L. plantarum* LBK treatment regulated these situations. In the H-LBK group, the expression level of GLUT9 was likewise reduced, when contrasted with the MOD group. We further observed that the expression level of URAT1 was reduced in contrast to the CON group under *L. plantarum* LBK and allopurinol regulation.

### 3.8 *Lactiplantibacillus plantarum* LBK reversed the abnormality of gut microbiota in hyperuricemia mice

**Figure 6:**
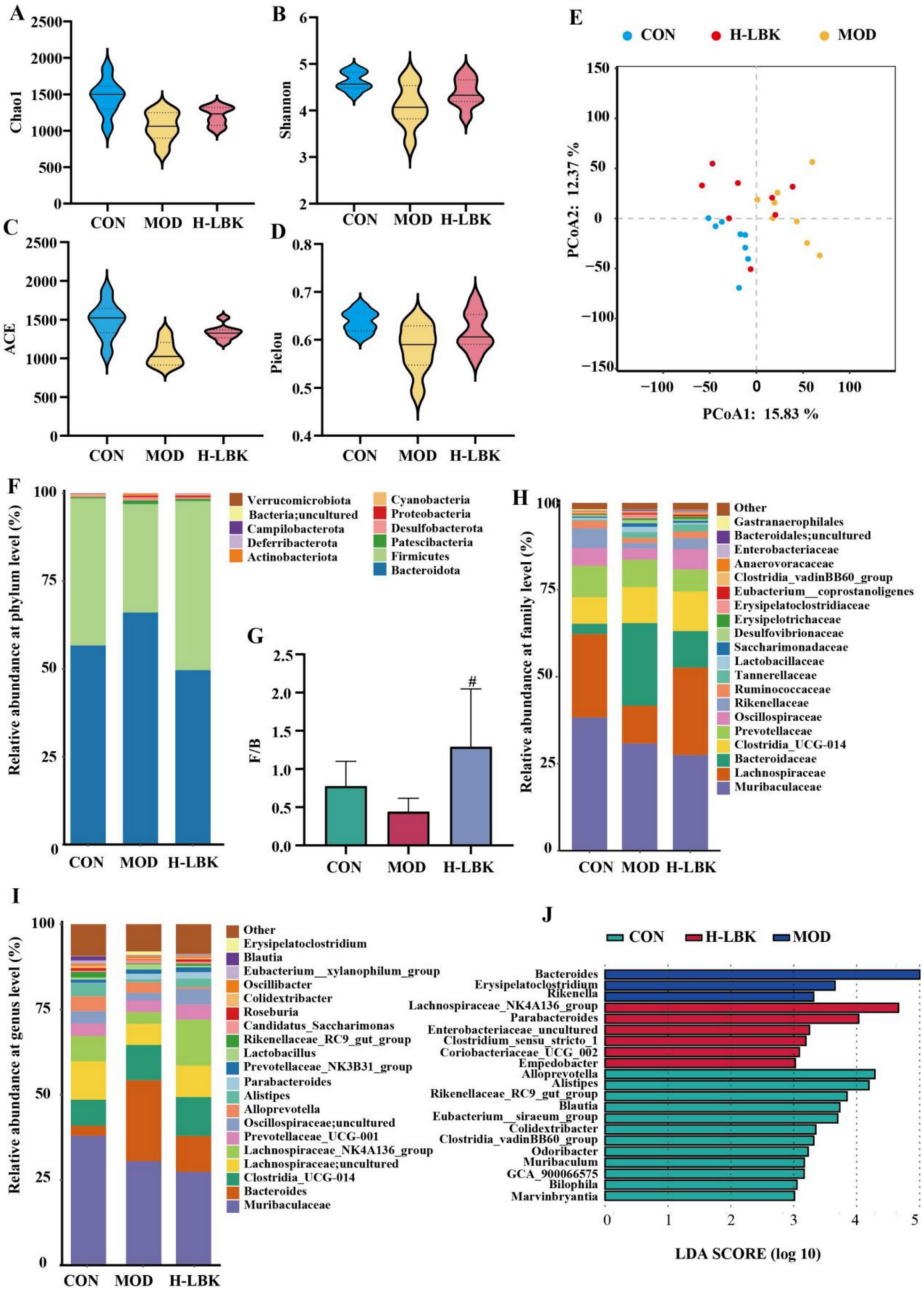
Effects of *L. plantarum* LBK on gut microbe of mice. **(A–D)** α-Diversity indexes of each group. **(E)** *β*-Diversity evaluated using the weighted UniFrac based PCoA. **(F)** Relative abundance of different bacteria at the phylum level. **(G)** Changes in the Firmicutes/Bacteroidetes ratio in the different groups. **(H)** Relative abundances of different bacteria at the family level. **(I)** Relative abundances of different bacteria at the genus level. **(J)** Linear discriminant analysis (LDA) effect size (LEfSe) method was used to investigate bacterial community at the genus level. LDA score higher than 3 indicates a higher relative abundance in the corresponding group than in other groups. #*p* < 0.05 vs. MOD group.

The richness and diversity of intestinal microbiota in mice were analyzed to study the therapeutic effect of *L. plantarum* LBK. Firstly, the α-diversity in mice was evaluated utilizing the Chao1, Shannon, ACE, and Pielou index. As shown in Figures 6A–D, Chao1, Shannon, ACE, and Pielou index reduced in the MOD group, while the decline was reversed in the H-LBK group. Furthermore, *β* diversity was estimated using principal coordinate analysis (PCoA) to determine the similarities between groups. As depicted in Figure 6E, the variance explained by the first principal coordinate axis of PCoA was found to be 15.83%, while that of the second principal coordinate was determined to be 12.37%. The microbiota between the CON and MOD groups was dissimilar. Hyperuricemia led to substantial alterations in the mouse microbiome structure, however, these changes were effectively mitigated upon the supplementation of *L. plantarum* LBK.

The effect of *L. plantarum* LBK on the composition of the gut microbiota was further investigated. At the phylum level, Bacteroidetes and Firmicutes represented the predominant microbiota within the mouse intestine (Figure 6F). The treatment of hyperuricemia in mice resulted in a significant increase in the relative abundance of Bacteroidetes and a concomitant decrease in Firmicutes. This was evidenced by a markedly lower Firmicutes/Bacteroidetes ratio (F/B ratio) observed in the MOD group compared to the CON group. Following the intervention by *L. plantarum* LBK, a notable elevation was observed in the F/B ratio of the H-LBK group (Figure 6G). At the family levels (Figure 6H), the dominant microbiota in the intestinal tract of mice were Muribaculaceae, lachnospiraceae, Bacteroidaceae, and Clostridia_UGG-014. At the genus levels (Figure 6I), there was a notable elevation in the relative abundance of *Bacteroides*, In the MOD group, the abundance of *lachnospiraceae_NK4A136_group* and *Lachnospiraceae_uncultured* decreased, whereas the administration of *L. plantarum* LBK significantly altered these shifts.

In addition, to investigate the specific microbes influenced by these groups, the LEfSe method (LDA > 3) was conducted (Figure 6J). The abundance of *Bacteroides*, *Erysipelatoclostridium,* and *Rikenella* increased significantly in the MOD group. In contrast, Oral *L. plantarum* LBK enhanced the abundance of *Lachnospiraceae_NK4A136_group* and *Parabacteroides*.

### 3.9 *Lactiplantibacillus plantarum* LBK altered fecal metabolites in HUA mice

**Figure 7:**
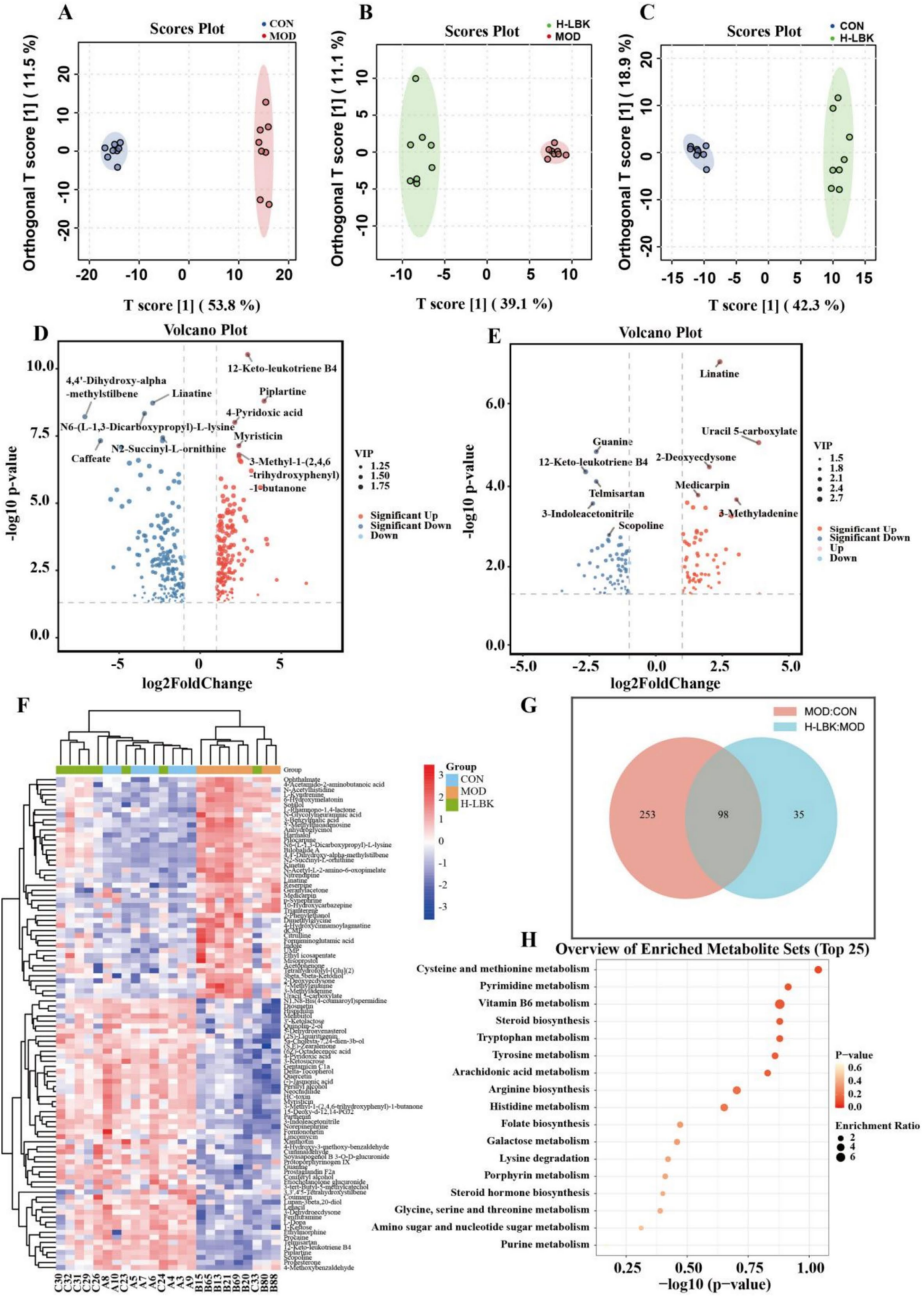
Metabolomic analysis of mice. **(A)** OPLS-DA score plot of CON group and MOD group. **(B)** OPLS-DA score plot of H-LBK group and MOD group. **(C)** OPLS-DA score plot of CON group and H-LBK group. **(D)** The volcano plot for metabonomic data of the MOD group and CON group. **(E)** The volcano plot for metabonomic data of the H-LBK group and MOD group. **(F)** Heatmap of differential metabolite (CON vs. MOD groups and MOD vs. H-LBK groups). **(G)** Veen diagram of differential metabolite (CON vs. MOD groups and MOD vs. H-LBK groups). **(H)** The metabolic pathway impact prediction between CON vs. MOD groups and MOD vs. H-LBK groups is based on the KEGG online database.

The fecal metabolites in the CON group, MOD group, and H-LBK group were investigated using nontargeted metabolomic analysis with the LC–MS/MS. To differentiate fecal metabolites, we utilized the Orthogonal Partial Least Squares Discriminant Analysis (OPLS-DA) method for individual group comparisons. OPLS-DA revealed that the overall fecal metabolic signatures of the samples were separated (Figures 7A–C). The differential metabolites were screened based on *p* < 0.05 and fold change ≥ 2 or fold change ≤ 0.5, and the result showed that 1,070 metabolites were identified. Compared with the CON group, a total of 352 metabolites were changed by HUA (Figure 7D). Furthermore, in comparison to MOD, 133 metabolites changed after the intervention of the *L. plantarum* LBK group (Figure 7E). Venn plots (Figure 7G) showed 98 differential metabolites in the two groups (CON vs. MOD and MOD vs. H-LBK). After normalization, a heatmap was generated to depict a total of 98 differential metabolites (Figure 7F). Notably, there was a downregulation of 54 metabolites (3-ketosucrose, 12-keto-leukotrieneB4, and piplartine) in the MOD group, however, as shown in Supplementary Table S3, the levels of 53 metabolites exhibited an inverse trend compared to the MOD group after *L. plantarum* LBK treatment.

To explore the potential metabolic pathways influenced by *L. plantarum* LBK, a KEGG enrichment analysis was conducted comparing the common differential metabolites between two groups (CON vs. MOD and MOD vs. H-LBK). As indicated in Figure 7H, functionally, these discriminative metabolites were primarily implicated in cysteine and methionine metabolism, pyrimidine metabolism, vitamin B6 metabolism, and tryptophan metabolism.

### 3.10 Analysis of the correlation between gut microbiota and fecal metabolome metabolites

The gut microbiota exerts a pivotal impact on the health of the host, primarily through the metabolites produced by microbiota. To elucidate the potential of *L. plantarum* LBK in mitigating hyperuricemia by modulating both the gut microbiota and its metabolic profile, we conducted Spearman’s correlation analysis to examine the relationships among distinct gut microbiota and their corresponding fecal microbiota metabolites.

**Figure 8:**
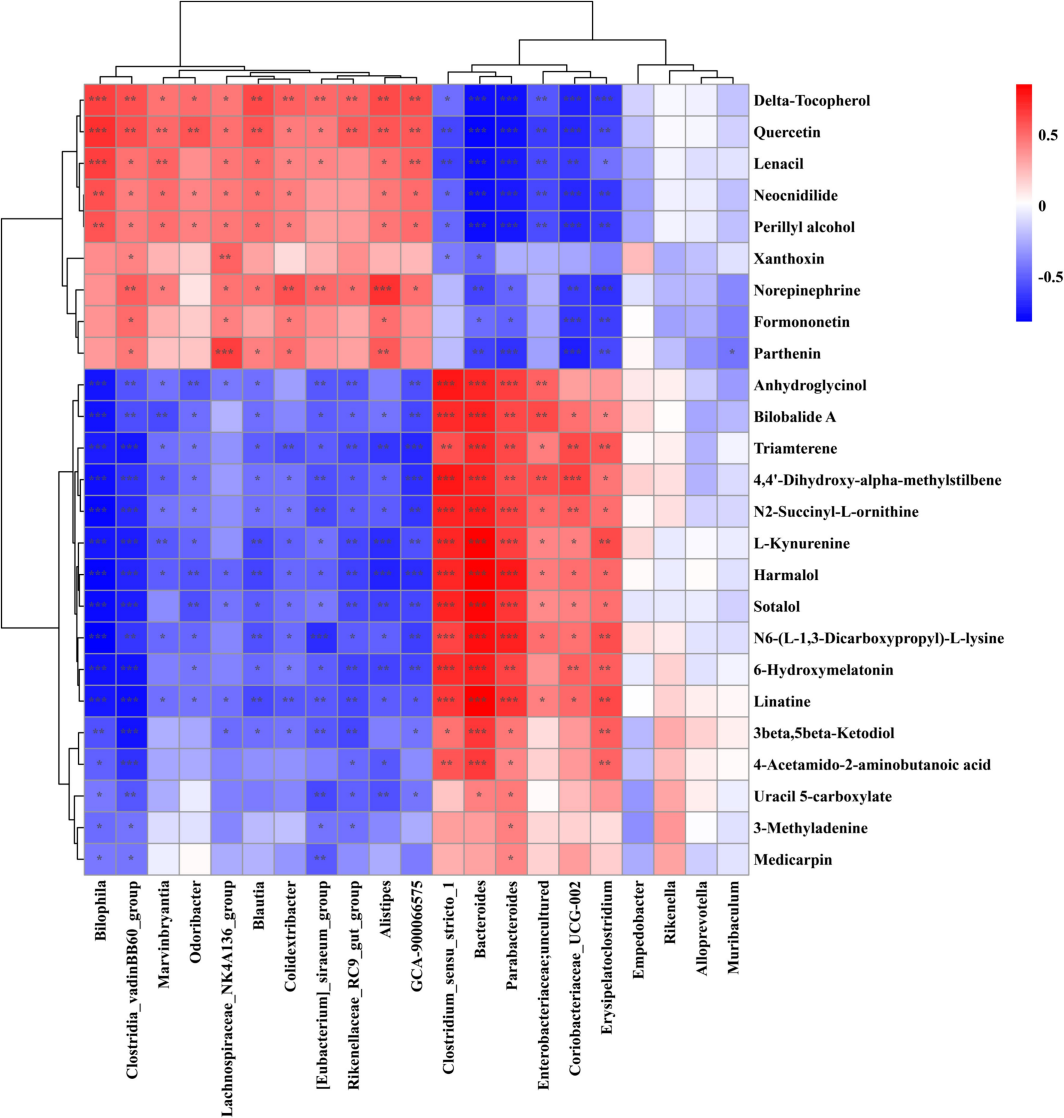
Heatmap of correlation between the key intestinal microbial phylotypes of significant differences and differential metabolites. *p < 0.05, **p < 0.01, ***p < 0.001.

The results revealed a significantly positive correlation between *Bacteroides* and anhydroglycinol, bilobalide A, triamterene, 4,4′-dihydroxy-alpha-methylstilbene and a strongly negative correlation with delta-tocopherol, quercetin, and lenacil. In addition, *Lachnospiraceae_NK4A136_group* was positively associated with parthenin and xanthoxin, and a strong negative correlation with the levels of linatine and 3beta, 5beta-ketodiol. Therefore, it was assumed that *L. plantarum* LBK may mitigate hyperuricemia through the modulation of intestinal flora and associated metabolites (Figure 8).

## 4 Discussion

Hyperuricemia is a metabolic disease that is associated with an increase in urate production and/or a decrease in urate excretion. This is due to various factors, including lifestyle, genetic predisposition, and impaired renal function. In the past few years, the prevalence of hyperuricemia has been on the rise continuously, and it has become a global problem in the healthcare system (Li et al., 2023). Therefore, it is significant to pay attention to and manage hyperuricemia. Purine is the precursor of uric acid, and purine metabolic disorder can induce hyperuricemia. Reducing purine intake can effectively control the source of uric acid. Probiotics are composed of living bacteria with various health effects, which are beneficial to human physiology and pathology and have become a potential natural therapy (Gareau et al., 2010). It is reported that fermented food rich in LAB is effective for many diseases.

Studies have reported the degradation of extracellular nucleosides or bases by lactic acid bacteria (Cao et al., 2022a; Cao et al., 2022b; Li, 2014; Lin et al., 2022), LAB can affect the circulating uric acid level. The ability of LAB to degrade nucleoside plays an important role in host uric acid metabolism. Therefore, in our study, inosine and guanosine were selected as the targets for screening probiotics. Among the candidate lactic acid bacteria screened from Koumiss, we found that 8 strains had good nucleoside degradation ability, among which 4 strains of LAB had degradation rates of inosine and guanosine as high as 90%. Similar degradation effects on purine nucleosides were observed for *Levilactobacillus brevis* grx821 (Wang et al., 2024). At the same time, it can be found that the degradation ability of the strain to inosine and guanosine is positively correlated, which may be because guanosine and guanosine can be degraded by the same enzyme, which indicates that the tested strain may contain inosine or guanosine hydrolase. Wang et al. (2019) reported that *Lactobacillus brevis* may degrade inosine through inosine hydrolase, thus improving microbial imbalance and lipid-sugar-related inflammation in mouse models. Similarly, *Lactobacillus paracasei* S12 (Xiao et al., 2020) from traditional kimchi can also degrade nucleoside *in vitro* and *in vivo*. The activity of xanthine oxidase can be inhibited to reduce the level of uric acid (Zhong et al., 2021). We tested the xanthine oxidase inhibitory ability of candidate lactic acid bacteria and found that all of them had certain inhibitory abilities. To assess the ability of the tested strains to survive and colonize successfully in the gastrointestinal environment, the biological characteristics of the candidate strains were evaluated. Candidate strain 2–1 showed better probiotic characteristics, which laid a foundation for exploring its potential to reduce uric acid in *vivo*. The similarity between strain 2–1 and *L. plantarum* was the highest (98.33%), and it was named *L. plantarum* LBK for animal experiments.

Based on this, we constructed a hyperuricemia mouse model to test the ability of *L. plantarum* LBK to alleviate hyperuricemia *in vivo*. Uric acid oxidase can degrade relatively insoluble UA into soluble allantoin, but in the long evolution process, uric acid oxidase was lost in humans and high primates due to the inactivation of genes encoding uricase. Therefore, we choose potassium oxalate (reducing uric acid production) and a high yeast diet (increasing uric acid source) to build the HUA model. Our research results confirmed that after 4 weeks of treatment, the organ index of the model group increased significantly, which was consistent with Cao (Cao et al., 2022a; Cao et al., 2022b). Compared with the model group, *L. plantarum* LBK has a certain effect of reducing UA in hyperuricemia mice, 10^9^ CFU/mL of *L. plantarum* LBK can reduce the serum UA to the normal range, which is better than that of 10^7^ CFU/mL in reducing UA in hyperuricemia model. Further analysis of the physiological and biochemical indexes in serum showed that it was consistent with the previous research results (Wu et al., 2021), after modeling, the indexes of BUN and CRE, were related to hyperuricemia, renal insufficiency, and liver function damage.

The kidney serves as the primary organ responsible for metabolizing, transporting, and excreting metabolic wastes. Previous studies have shown that UA can accumulate in the kidney and induce chronic renal injury, which is accompanied by an increase in CRE, BUN, and other biomarkers. The greater the concentration of these markers are, the more severe the resulting injury will be (Wei et al., 2021). However, after *L. plantarum* LBK treatment by *Lactobacillus plantarum*, these indexes were adjusted to normal levels. This may be due to the decrease of hepatic urea production and the increase of renal uric acid clearance rate and contribute to the recovery of renal damage. XOD can catalyze hypoxanthine and xanthine to produce uric acid (Li H. et al., 2020; Li F. et al., 2020). These are the last two steps of urate biosynthesis, the inhibition of XOD is an effective way to treat HUA (Zhang et al., 2018). Following the administration of *L. plantarum* LBK, the increase in XOD activity in the liver returned to normal levels, in accordance with the findings of *in vitro* experiments, suggesting that *L. plantarum* LBK may hinder the production of uric acid and lead to liver protective activity against HUA treatment. Wang (Wang et al., 2023) found that *Bacillus subtilis*-fermented *Astragalus membranaceus* can also significantly reduce the XOD activity in the liver of hyperuricemia mice. A similar phenomenon was also detected in H&E observation. Compared with MOD, the ALLO group has a further injury, while the *L. plantarum* LBK group has an alleviation effect, which shows that *L. plantarum* LBK can mitigate the damage to the liver and kidney in mice and circumvent the adverse effects associated with ALLO. Although *L. plantarum* LBK treatment reversed the injury caused by HUA, inflammatory cell infiltration could still be found in renal tissue. Then, we detected cytokines associated with inflammation. We detected the up-regulation of inflammatory cytokines such as IL-6 and TNF-*α* in the liver and kidney of hyperuricemia mice induced by potassium oxalate and a high yeast diet. After the treatment of *L. plantarum* LBK, the abnormalities of these inflammatory cytokines were effectively alleviated, suggesting that *L. plantarum* LBK may have an anti-inflammatory effect on HUA.

After forming in the liver, two thirds of the UA is excreted through the kidneys (Kielstein et al., 2020). Following glomerular filtration, uric acid is secreted and reabsorbed in the proximal tubules of the kidney via the uric acid transporter protein. ABCG2 is a UA secretion transporter, which can transport UA from blood to urine. The malfunction of this system can result in a reduction in uric acid excretion, which in turn can lead to an increase in blood uric acid levels (Ichida, 2014). The uric acid transporters GLUT9 and URAT1 are primarily associated with uric acid reabsorption. After *L. plantarum* LBK treatment, the expression of ABCG2 in the kidney was found to be elevated, while the expression of uric acid reuptake transporters GLUT9 and URAT1 was observed to be reduced. At present, researchers have found that some probiotics affect the expression of UA transporter in hyperuricemia, The expression of ABCG2 at mRNA level was up-regulated by *L. rhamnosus* Fmb14 (Zhao et al., 2023), and the biosynthesis of GLUT9 was reduced. Similarly, *L. paracasei* MJM60396 (Lee et al., 2022) inhibited the reabsorption of uric acid by reducing the expression of URAT1 and GLUT9. In addition, HUA can make the transporters of ABCG2 and GLUT9 in the intestine out of balance, and obviously reduce the excretion of uric acid in the intestine (Yin et al., 2022). Although probiotics can regulate the expression of UA transporter to reduce uric acid levels, its mechanism of regulating UA transporter expression level needs further study.

The emergence and progression of hyperuricemia are closely related to intestinal microflora and its metabolites. Patients with HUA had decreased microbial diversity and unbalanced intestinal microecology (Wei et al., 2022), the administration of *L. plantarum* LBK resulted in a moderate restoration of the diversity and abundance of intestinal flora. The *L. plantarum* LBK can effectively alleviate the decline in the relative abundance of beneficial bacteria resulting from the HUA model. F/B ratio seems to be related to relieving hyperuricemia, pretreatment of *L. fermentum* F40-4 (Cao et al., 2023) and JL-3 (Wei B. et al., 2022; Wei J. et al., 2022) significantly increased the number of Firmicutes, decreased the number of Bacteroides and ameliorated hyperuricemia. *Lachnospiraceae_NK4A136_group* is the dominant microorganism in the H-LBK group, and its abundance decreased in the MOD group, after gavaged, its abundance increased. In the high-fat diet mice, the abundance of *Lachnospiraceae_NK4A136_group* was decreased (Li et al., 2020), which suggested that it may be a potential probiotic, but its function was not clear. The UA will be metabolized by the microflora in the intestine after it is secreted into the intestine. Metabolites derived from tryptophan metabolism may play biological roles. Our investigation revealed that the levels of 3-indoleacetonitrile were elevated following treatment with *L. plantarum* LBK, and we hypothesized that *L. plantarum* LBK could regulate the content of small molecule metabolites in tryptophan metabolism and ameliorate hyperuricemia. *Erysipelatoclostridium*, a pathogenic bacterium, its relative abundance was increased in the MOD group. Spearman analysis revealed a positive correlation between the two variables, with a significant association observed between l-kynurenine content and the parameter of interest (*p* < 0.05). l-kynurenine is the product of tryptophan metabolism, and the increase of tryptophan metabolite level will increase the level of inflammation in the body, thus causing adverse effects (Shi et al., 2020). Alterations in the intestinal flora were predominantly linked to biomarkers including amino acid metabolism, pyrimidine metabolism, and steroid biosynthesis. It is speculated that *L. plantarum* LBK may indirectly play a role in regulating amino acid metabolism *in vivo* by regulating intestinal flora, thus intervening in HUA.

Our results revealed that *Lactiplantibacillus plantarum* LBK alleviates hyperuricemia through modulation of the gut microbiota, as demonstrated by integrated 16S rRNA gene sequencing and untargeted metabolomics analysis. While 16S rRNA gene sequencing effectively profiles community composition from phylum to genus, its limited resolution at the species and strain levels may prevent the identification of functionally distinct taxa. Concurrently, the untargeted metabolomics data offer relative, not absolute, quantification of metabolite abundances. Consequently, the functional implications of these findings necessitate future validation using targeted metabolomics.

## 5 Conclusion

In conclusion, our findings focused on a novel strain of *L. plantarum* LBK from Koumiss. This strain not only degraded inosine and guanosine *in vitro* but also alleviated hyperuricemia *in vivo*. Specifically, *L. plantarum* LBK degraded inosine and guanosine, inhibited XOD activity, modulated renal uric acid transporter protein expression, mitigated inflammation, and ameliorated renal injury. Furthermore, *L. plantarum* LBK could alleviate hyperuricemia by modulating gut microbiota and intestinal metabolites. Therefore, *L. plantarum* LBK holds promise as a potential probiotic for the improvement of hyperuricemia. Further studies are needed to evaluate the safety and efficacy in human applications.

## Data Availability

All relevant data is contained within the article: The original contributions presented in the study are included in the article/Supplementary material, further inquiries can be directed to the corresponding author/s.

## Footnotes

1
http://www.hmdb.ca/

2
https://metlin.scripps.edu/

3
https://cloud.majorbio.com

4
https://www.kegg.jp/kegg/pathway.html
