# Supplementary material for: Probiotic potential of Lactiplantibacillus plantarum LBK from Koumiss in reducing hyperuricemia through gut microbiota modulation
Source: Front Microbiol. 2026 May 20;16:1716437. doi: 10.3389/fmicb.2025.1716437 (PMC13229888; doi:10.3389/fmicb.2025.1716437)
Supplement: Supplementary file 1 [file Table_1.DOCX]

**Appendix A. Supplementary materials**

**Supplementary Table 1**

Primer sequences for ABCG2、GLUT9、URAT1 and GAPDH genes

| Gene | Primer | Gene primer sequence |
| --- | --- | --- |
| ABCG2 | Forward | 5'-TAGGACGCTCGCAGAAGGA-3' |
|  | Reverse | 5'-AGAATAGCATTAAGGCCAGGTTT-3' |
| GLUT9 | Forward | 5'-ATGTGGACTCAATGCGATCTGGTTC-3' |
|  | Reverse | 5'-TGTTTCAATTCCTCCCGTGCTCAG-3' |
| URAT1 | Forward | 5'-GACCTTGGACCCGATGTTCTTCTG-3' |
|  | Reverse | 5'-CGTGGCGTTGGACTCTGTAAGC-3' |
| GAPDH | Forward | 5'-AGGTCGGTGTGAACGGATTTG-3' |
|  | Reverse | 5'-TGTAGACCATGTAGTTGAGGTCA-3' |

**Supplementary Table 2**

Degradation rate of inosine and guanosine

| Serial number | | Strain number | Degradation rate of inosine（%） | Degradation rate of guanosine（%） |
| --- | --- | --- | --- | --- |
| 1 | 11-1 | | 22.59 ± 0.04 | 40.68 ± 0.15 |
| 2 | 11-3 | | 52.9 ± 2.32 | 71.03 ± 3.01 |
| 3 | 11-4 | | 9.43 ± 0.14 | 15.06 ± 0.12 |
| 4 | 11-5 | | 13.38 ± 0.08 | 39.24 ± 0.14 |
| 5 | 11-7 | | 19.92 ± 0.05 | 48.5 ± 0.10 |
| 6 | 12-1 | | 4.07 ± 0.08 | 2.79 ± 0.11 |
| 7 | 12-2 | | 5.10 ± 0.12 | 6.01 ± 0.09 |
| 8 | 12-4 | | 5.01 ± 0.02 | 1.58 ± 0.11 |
| 9 | 13-1 | | 11.95 ± 0.04 | 18.75 ± 0.1 |
| 10 | 13-12 | | 1.29 ± 0.23 | 8.71 ± 0.18 |
| 11 | 13-13 | | 4.66 ± 0.10 | 9.02 ± 0.09 |
| 12 | 13-14 | | 0.69 ± 0.09 | 8.17 ± 0.06 |
| 13 | 13-15 | | 1.60 ± 0.06 | 14.86 ± 0.17 |
| 14 | 13-17 | | 2.86 ± 0.09 | 16.60 ± 0.12 |
| 15 | 13-18 | | 2.72 ± 0.17 | 4.62 ± 0.15 |
| 16 | 13-3 | | 6.44 ± 0.07 | 2.83 ± 0.07 |
| 17 | 13-5 | | 4.60 ± 0.04 | 2.60 ± 0.05 |
| 18 | 13-7 | | 5.74 ± 0.13 | 10.23 ± 0.06 |
| 19 | 13-8 | | 1.29 ± 0.07 | 11.54 ± 0.06 |
| 20 | 13-9 | | 4.82 ± 0.11 | 10.10 ± 0.13 |
| 21 | 15-1 | | 5.00 ± 0.23 | 3.83 ± 0.06 |
| 22 | 15-15 | | 10.06 ± 0.09 | 11.99 ± 0.61 |
| 23 | 15-18 | | 0.54 ± 0.05 | 16.27 ± 0.13 |
| 24 | 15-19 | | 7.33 ± 0.09 | 21.52 ± 0.08 |
| 25 | 15-2 | | 1.68 ± 0.07 | 17.44 ± 0.11 |
| 26 | 15-3 | | 3.81 ± 0.04 | 2.82 ± 0.07 |
| 27 | 15-4 | | 5.46 ± 0.08 | 6.31 ± 0.10 |
| 28 | 15-5 | | 6.21 ± 0.09 | 6.25 ± 0.03 |
| 29 | 15-6 | | 5.24 ± 0.03 | 3.32 ± 0.06 |
| 30 | 15-7 | | 4.32 ± 0.03 | 2.53 ± 0.05 |
| 31 | 15-8 | | 5.96 ± 0.08 | 11.17 ± 0.15 |
| 32 | 25-1 | | 7.47 ± 0.07 | 8.45 ± 0.19 |
| 33 | 25-12 | | 0.75 ± 0.03 | 6.30 ± 0.12 |
| 34 | 25-14 | | 73.85 ± 0.4 | 90.16 ± 0.06 |
| 35 | 25-16 | | 9.53 ± 0.19 | 9.12 ± 0.10 |
| 36 | 25-17 | | 7.14 ± 0.05 | 10.01 ± 0.07 |
| 37 | 25-18 | | 0.17 ± 0.05 | 12.59 ± 0.26 |
| 38 | 25-2 | | 4.31 ± 0.06 | 15.10 ± 0.11 |
| 39 | 25-20 | | 6.27 ± 0.05 | 7.92 ± 0.03 |
| 40 | 25-3 | | 7.05 ± 0.06 | 9.54 ± 0.18 |
| 41 | 25-4 | | 11.16 ± 0.12 | 29.33 ± 0.12 |
| 42 | 25-5 | | 3.36 ± 0.07 | 3.22 ± 0.22 |
| 43 | 25-6 | | 5.44 ± 0.14 | 14.16 ± 0.16 |
| 44 | 25-7 | | 2.16 ± 0.05 | 0.40 ± 0.08 |
| 45 | 25-8 | | 9.82 ± 0.18 | 26.14 ± 0.11 |
| 46 | 29B | | 100.00 ± 0.00 | 100.00 ± 0.00 |
| 47 | 32-2 | | 9.02 ± 0.10 | 10.60 ± 0.17 |
| 48 | 36-1 | | 7.21 ± 0.04 | 9.68 ± 0.14 |
| 49 | 36-2 | | 8.05 ± 0.07 | 8.70 ±0.27 |
| 50 | 4-1 | | 7.21 ± 0.03 | 7.19 ± 0.21 |
| 51 | 4-2 | | 8.39 ± 0.14 | 25.53 ± 0.45 |
| 52 | 4-4 | | 7.51 ± 0.07 | 24.43 ± 0.12 |
| 53 | 4-6 | | 7.58 ± 0.12 | 27.13 ± 0.24 |
| 54 | 53-1 | | 7.07 ± 0.05 | 10.69 ± 0.16 |
| 55 | 54-1 | | 7.78 ± 0.14 | 8.75 ± 0.21 |
| 56 | 55-2 | | 9.47 ± 0.05 | 10.34 ± 0.12 |
| 57 | 9A | | 100.00 ± 0.00 | 100.00 ± 0.00 |
| 58 | 1M1 | | 31.82 ± 0.16 | 32.91 ± 2.05 |
| 59 | 1M13 | | 41.59 ± 0.14 | 45.24 ± 0.14 |
| 60 | 1M2 | | 33.4 ± 0.16 | 35.27 ± 0.12 |
| 61 | 1M21 | | 34.8 ± 0.21 | 36.79 ± 0.18 |
| 62 | 1M22 | | 37.9 ± 0.38 | 40.96 ± 0.24 |
| 63 | 1M23 | | 45.42 ± 1.15 | 48.19 ± 1.22 |
| 64 | 1M32 | | 41.30 ± 0.11 | 54.76 ± 0.37 |
| 65 | 1M33 | | 34.90 ± 0.11 | 34.66 ± 0.12 |
| 66 | 1M7 | | 33.83 ± 0.06 | 33.46 ± 0.11 |
| 67 | 3M73 | | 6.49 ± 0.06 | 10.96 ± 0.04 |
| 68 | 5M26 | | 28.43 ± 0.12 | 27.58 ± 0.37 |
| 70 | 1-2 | | 95.38 ± 3.92 | 98.62 ± 1.28 |
| 71 | 1-3 | | 100.00 ± 0.00 | 100.00 ± 0.00 |
| 72 | 2-1 | | 91.16 ± 0.54 | 96.51 ± 0.45 |
| 73 | 3-2 | | 4.08 ± 0.12 | 5.77 ± 0.10 |

**Supplementary Table 3**

Metabolites altered by *L. plantarum* LBK

| Serial number | Metabolites | MOD | H-LBK |
| --- | --- | --- | --- |
| 1 | 3-Ketosucrose | ↓ | ↑ |
| 2 | 12-Keto-leukotriene B4 | ↓ | ↑ |
| 3 | Piplartine | ↓ | ↑ |
| 4 | 4-Pyridoxic acid | ↓ | ↑ |
| 5 | Myristicin | ↓ | ↑ |
| 6 | Scopoline | ↓ | ↑ |
| 7 | 3-Methyl-1-(2,4,6-trihydroxyphenyl)-1-butanone | ↓ | ↑ |
| 8 | 3-Indoleacetonitrile | ↓ | ↑ |
| 9 | Delta-Tocopherol | ↓ | ↑ |
| 10 | (6Z)-Octadecenoic acid | ↓ | ↑ |
| 11 | 1-Kestose | ↓ | ↑ |
| 12 | L-Dopa | ↓ | ↑ |
| 13 | 3'-Ketolactose | ↓ | ↑ |
| 14 | Gentamicin C1a | ↓ | ↑ |
| 15 | Fenfluramine | ↓ | ↑ |
| 16 | Quercetin | ↓ | ↑ |
| 17 | Lenacil | ↓ | ↑ |
| 18 | Melibiitol | ↓ | ↑ |
| 19 | Telmisartan | ↓ | ↑ |
| 20 | Guanine | ↓ | ↑ |
| 21 | Neocnidilide | ↓ | ↑ |
| 22 | 3-Dehydroecdysone | ↓ | ↑ |
| 23 | Coumarin | ↓ | ↑ |
| 24 | (-)-Jasmonic acid | ↓ | ↑ |
| 25 | Norepinephrine | ↓ | ↑ |
| 26 | Perillyl alcohol | ↓ | ↑ |
| 27 | Coniferyl alcohol | ↓ | ↑ |
| 28 | 15-Deoxy-d-12,14-PGJ2 | ↓ | ↑ |
| 29 | Parthenin | ↓ | ↑ |
| 30 | Lupan-3beta,20-diol | ↓ | ↑ |
| 31 | Diosmetin | ↓ | ↑ |
| 32 | N1, N8-Bis(4-coumaroyl) spermidine | ↓ | ↑ |
| 33 | Procaine | ↓ | ↑ |
| 34 | Soyasapogenol B 3-O-D-glucuronide | ↓ | ↑ |
| 35 | Hispidulin | ↓ | ↑ |
| 36 | HC-toxin | ↓ | ↑ |
| 37 | 4-Methoxybenzaldehyde | ↓ | ↑ |
| 38 | Prostaglandin F2a | ↓ | ↑ |
| 39 | Quinolin-2-ol | ↓ | ↑ |
| 40 | 4-Hydroxy-3-methoxy-benzaldehyde | ↓ | ↑ |
| 41 | Cuminaldehyde | ↓ | ↑ |
| 42 | 5a-Cholesta-7,24-dien-3b-ol | ↓ | ↑ |
| 43 | 5-Dehydroavenasterol | ↓ | ↑ |
| 44 | Progesterone | ↓ | ↑ |
| 45 | (S, E)-Zearalenone | ↓ | ↑ |
| 46 | Lincomycin | ↓ | ↑ |
| 47 | Xanthoxin | ↓ | ↑ |
| 48 | Formononetin | ↓ | ↑ |
| 49 | 3-tert-Butyl-5-methylcatechol | ↓ | ↑ |
| 50 | Etiocholanolone glucuronide | ↓ | ↑ |
| 51 | (2S)-Liquiritigenin | ↓ | ↑ |
| 52 | 3,3',4'5-Tetrahydroxystilbene | ↓ | ↓ |
| 53 | Ethylmorphine | ↓ | ↑ |
| 54 | Protoporphyrinogen IX | ↓ | ↑ |
